# Supplementary material for: A One-Year Retrospective Analysis of Viral and Parasitological Agents in Wildlife Animals Admitted to a First Aid Hospital
Source: Animals (Basel). 2023 Mar 4;13(5):931. doi: 10.3390/ani13050931 (PMC10000059; doi:10.3390/ani13050931)
Supplement: Supplementary file 1 [file animals-13-00931-s001.zip › animals-2228219-supplementary-done.pdf]

SUPPLEMENTARY DATA

Table S1. Summary of the specie-specific panels for virological molecular investigations.

	ROE DEER AND FALLOW DEER	FOX	BADGER AND PINE MARTEN	PORCUPINE
**BOCAVIRUS**	BoV spp	CBoV 1<br>CBoV 2<br>CBoV 3<br>BoV spp	CBoV 1<br>CBoV 2<br>CBoV 3<br>BoV spp	BoV spp
**PARVOVIRUS**	--	CPV<br>FPV	CPV<br>FPV	--
**ADENOVIRUS**	--	CAdV 1<br>CAdv 2	CAdV 1<br>CAdv 2	--
**TORQUE TENO VIRUS**	--	TTV1<br>TTV2	TTV1<br>TTV2	--
**PESTIVIRUS**	BVDV<br>Pestivirus spp	--	--	--
**MORBILLIVIRUS**	--	CDV	CDV	--
**BOPIVIRUS**	BopV spp	--	--	--
**KOBUVIRUS**	KoV spp	KoV spp	KoV spp	KoV spp
**CORONAVIRUS**	CoV spp	CoV spp	CoV spp	CoV spp
**ASTROVIRUS**	AstV spp	AstV spp	AstV spp	AstV spp

Table S2. The primer sets used in the study.

	PRIMERS SEQUENCES	MELTING T° (°C)	AMPLION LENGTH (bp)	REFERENCE
**CBoV 1**	Fw1: CARTGGTAYGCTCCMATYTTTAA<br>Rw1: TGGCTCCCGTCACAAAAKATRTG<br>Fw2: TGGTAYGCTCCMATYTTTAAYGG<br>Rw2: GCTCCCGTCACAAAAKATRTGAAC	55	400	Kapoor et al., 2012
**CBoV 2**	Fw: AGGTCGGCCACTGGCTGT<br>Rw: CAGCTTAACGGCATTCACTA	55	128	Lau et al., 2012
**CBoV 3**	Fw1: CAGATTTGGGGGTCCTGCAT<br>Rw1: GCACTGTCTGCGCTGAAAAA<br>Fw2: ATGCCGTCACCAATCCACAT<br>Rw2: AGCTTGTGGTGGACAGTAGC	55	173	Li et al., 2013
**BoV spp**	Fw: GCCAGCACNGGNAARACMAA<br>Rw: CATNAGNCAYTCYTCCCACCA	55	141	Lau et al., 2012
**CPV**	Fw: ACAAGATAAAAGACGTGGTGTAACTCAA<br>Rw: CAACCTCAGCTGGTCTCATAATAGT	55	193	Schatzberg et al., 2003
**FPV/CPV**	Fw: ACAAGATAAAAGACGTGGTGTAACTCAA<br>Rw: CAACCTCAGCTGGTCTCATAATAGT	55	83	Ndiana et al., 2021
**CAdV**	Fw: CGCGCTGAACATTACTACCTTGTC<br>Rw: CCTAGAGCACTTCGTGTCCGCTT	58	CAdV-1: 508<br>CAdV-2: 1030	Hu et al., 2001
**TTV 1**	Fw: CGGGTTCAGGAGGCTCAAT<br>Rw: GCCATTCGGAACTGCACTTACT	55	305	Segales et al., 2009
**TTV 2**	Fw: TCATGACAGGGTTCACCGGA<br>Rw: CGTCTGCGCACTTACTTATATACTCTA	55	252	Segales et al., 2009
**BVDV**	Fw: ATGCCCWTAGTAGGACTAGCA	53	288	Vilcek et al., 1994

	Rw: TCAACTCCATGTGCCATGTAC			
**Pestivirus spp**	Fw: GATGCCATGTGGACGAGGGC<br>Rw: CATGTGCCATGTACAGCAGAG	55	150	Goto et al., 2021
**CDV**	Fw: ACTTCCGCGATCTCCACTGG<br>Rw: GCTCCACTGCATCTGTATGG	60	177	Conceição-Neto, 2017
**BopV spp**	Fw: CTGRGCAAGTTCACCAACAA<br>Rw: GTCCATGACAGGGTGAATCA	52	627	Laszlo et al., 2021
**KoV spp**	Fw: TGGAYTACAAGTGTTTTGATGC<br>Rw: ATGTTGTTRATGATGGTGTTGA	53	216	Reuter 2009
**CoV spp**	Fw1: GGKTGGGAYTAYCCKAARTG<br>Rw1: TGYTGTSWRCARAAYTCRTG<br>Fw2: GGTTGGGACTATCCTAAGTGTGA<br>Rw2: CCATCATCAGATAGAATCATCAT	48	440	Chu et al., 2011
**AstV spp**	Fw1a: GARTTYGATTGGRCKCGKTAYGA<br>Fw1b: GARTTYGATTGGRCKAGGTAYGA<br>Fw2a: CGKTAYGATGGKACKATHCC<br>Fw2b: AGGTAYGATGGKACKATHCC<br>Rw: GGYTTKACCCACATNCCRAA	50	422	Chu et al., 2008

Table S3. Accession number of the sequences entered on the GenBank database and results of their analysis with the Blast software.

	Species	GenBank Accession Number	Sequence Length	Reference Sequence	Percent Identity	E Value
**CAdV1**	Fox	CAdV/fox/894/IT<br>OP851367	497	Y07760.1	99,8%	0.0
	Badger	CAdV/badger/153/IT<br>OP851366	497	Y07760.1	99,6%	0.0
**KoV**	Roe deer	kov/roedeer/149/IT<br>OP851362	218	KF781169.1	96,33%	$1e^{-94}$
	Fox	kov/fox/580/IT<br>OP851364	193	JN387133.1	94,33%	$8e^{-76}$
	Badger	Kov/badger/540/IT<br>OP851365	233	KF781169.1	92,86%	$2e^{-78}$
	Porcupine	kov/porcupine/1243/IT<br>OP851363	218	KF781169.1	96,79%	2e-96
**AstV**	Roe deer	Mastv/roedeer/32/IT<br>OP851369	344	MK004956.1	74,24%	$1e^{-11}$
	Fox	Mastv/fox/1195/IT<br>OP851368	362	MW504559.1	75,42%	$1e^{-17}$
**BopV**	Fallow deer	Bopv/fallowdeer/675/IT<br>OP851370	562	MZ436972.1	94,66%	0.0
